# Immune Repertoire after Immunization As Seen by Next-Generation Sequencing and Proteomics

**DOI:** 10.3389/fimmu.2017.01286

**Published:** 2017-10-16

**Authors:** Martijn M. VanDuijn, Lennard J. Dekker, Wilfred F. J. van IJcken, Peter A. E. Sillevis Smitt, Theo M. Luider

**Affiliations:** ^1^Department of Neurology, Erasmus MC, Rotterdam, Netherlands; ^2^Erasmus Center for Biomics, Erasmus MC, Rotterdam, Netherlands

**Keywords:** immune repertoire, immunization, NGS, mass spectrometry, immunoglobulins

## Abstract

The immune system produces a diverse repertoire of immunoglobulins in response to foreign antigens. During B-cell development, VDJ recombination and somatic mutations generate diversity, whereas selection processes remove it. Using both proteomic and NGS approaches, we characterized the immune repertoires in groups of rats after immunization with purified antigens. Proteomics and NGS data on the repertoire are in qualitative agreement, but did show quantitative differences that may relate to differences between the biological niches that were sampled for these approaches. Both methods contributed complementary information in the characterization of the immune repertoire. It was found that the immune repertoires resulting from each antigen had many similarities that allowed samples to cluster together, and that mutated immunoglobulin peptides were shared among animals with a response to the same antigen significantly more than for different antigens. However, the number of shared sequences decreased in a log-linear fashion relative to the number of animals that share them, which may affect future applications. A phylogenetic analysis on the NGS reads showed that reads from different individuals immunized with the same antigen populated distinct branches of the phylogram, an indication that the repertoire had converged. Also, similar mutation patterns were found in branches of the phylogenetic tree that were associated with antigen-specific immunoglobulins through proteomics data. Thus, data from different analysis methods and different experimental platforms show that the immunoglobulin repertoires of immunized animals have overlapping and converging features. With additional research, this may enable interesting applications in biotechnology and clinical diagnostics.

## Introduction

The basic understanding of the molecular biology that leads to diversity in the adaptive immune response emerged around 1980 (1), an effort that was awarded with a Nobel prize for Physiology and Medicine for Tonegawa. Yet, it is only in recent years that technology has advanced sufficiently to study the population of sequences that results from this recombination process and the subsequent mutation and selection pressures for the formation of mature immunoglobulins (2). The high-throughput sequencing methods that are available allow researchers to obtain a listing of the repertoire of sequences that make up the antibodies or T-cell receptors that mediate the adaptive immune response. Research groups have started using and refining such tools to understand the development of immune responses, and envision potential applications of information on the immune repertoire.

Yet, it is challenging to obtain a sample for sequencing that properly reflects the repertoire of antibody proteins that is present in the serum, and even more the repertoire of an antigen-specific subset of sequences. One challenge is that not all cells with a rearranged immunoglobulin locus express immunoglobulin protein. Distinctions have been found between the B-cell receptor repertoire and the plasma cell repertoire that drives immunoglobulin expression (3). Another challenge is the tissue niche that is sampled for obtaining sequence information. The immune response is a compartmentalized process that takes place in circulating blood, in the interstitial space of (inflamed) tissues, and in lymphoid organs, such as lymph nodes, the spleen, or bone marrow. The timing and location of the sampling sites are likely to affect the immune repertoire that is observed, and not all sites are easily accessible, especially in human subjects. However, antibodies that are produced as a result of an immune response will generally end up in the circulation regardless of the site of production. Antibody proteins can be collected from serum and affinity-enriched in order to study an antigen-specific subset of molecules. For these reasons, we here study the immune repertoire with a combination of proteomics and NGS. In this way, we can obtain a more comprehensive picture of the differences but also similarities that exist between individuals after an immune response to a particular antigen. The techniques were already combined successfully in the past, and can help provide unique but not always consistent views on the repertoire (4–8).

We previously found evidence for common features between antigen-specific immune sera. The findings are consistent with an increasing body of literature that reports commonalities in the sequence of immunoglobulins targeting a particular antigen (9–14). An immune repertoire consisting of sequences that are not unique to an individual is referred to as a public or stereotyped response. It is thought that such responses result from the selection of specific rearrangements during the initial immune response, or the selection of similar somatic mutations through a process of convergent evolution of the repertoire.

This experiment was designed with a number of distinguishing characteristics that define the data that were collected. First, the immune repertoire was studied in a group of laboratory outbred animals rather than in a heterogeneous population of human subjects. Second, the animals were all immunized with a purified antigen rather than with a pathogen that exposes a multitude of antigens and epitopes. Finally, the samples were analyzed with a combination of proteomics and long-read NGS, two techniques that provide complementary on the immune repertories and both allow us to examine the entire variable domain of the immunoglobulins. With proteomics, affinity-enriched antibodies can be studied, but with limited sequence length or sequence accuracy. Our NGS method offers superior depth, read length, and sequence accuracy, and in combination the strengths of both can be combined. With this dataset, we aim to validate our earlier proteomics observations on convergence in antigen-specific immune repertoires, perform an extended analysis with the NGS data, and establish the value of both techniques in the study of immune repertoires.

## Materials and Methods

Wistar rats were immunized and analyzed by proteomics as described earlier, and spleen material collected from these animals is now used for NGS analysis (10). Rat immunization and sample collection was performed by Genovac GmbH (Freiburg, Germany) under their local permits and regulatory framework. The immunization and three boosts were performed with either recombinant HuD or Keyhole Limpet Hemocyanine modified with dinitrophenyl (DNP) residues, each time with 2-week intervals. HuD is an onconeural antigen related to a paraneoplastic neurological syndrome (15), and DNP was chosen as a well-defined small epitope. Pre-immune and immune sera were collected, as well as a spleen cell suspension. IgG was isolated from the sera with Melon Gel (Invitrogen, Carlsbad, CA, USA), optionally affinity enriched against HuD or DNP-ovalbumin immobilized on Aminolink Plus particles (Thermo Fisher Scientific, Rockford, IL, USA), digested with trypsin and analyzed by a 90 min gradient on a Pepmap Acclaim column, coupled to on an LTQ Orbitrap XL (Thermo Fisher Scientific, Bremen, Germany) set at 30,000 resolution MS1 in the Orbitrap and using dynamic data acquisition to produce CID MS–MS spectra in the ion trap.

For NGS, RNA was collected from 50 × 10^6^ total splenocytes using Trizol (Life Technologies) and additional cleanup with an RNeasy spin column (Qiagen, Germantown, MD, USA). 5 μg RNA collected from splenocytes was reverse transcribed to cDNA with Superscript III (Invitrogen, Waltham, MA, USA) and primers complementary to the constant domains, which included a Unique Molecular Identifier segment (UMI; Supplementary Material). After the addition of Superscript III, reverse transcription proceeded for 40 min at 50°C, after which the enzyme was inactivated at 70°C for 15 min. After cDNA product cleanup with AmpureXP beads (Beckman Coulter, Indianapolis IN, USA), PCR was performed with Phusion proofreading polymerase in HighFidelity buffer (New England Biolabs, Ipswich, MA, USA). The cDNA was amplified with a multiplex degenerate forward primer set and a common reverse primer. Forward PCR primers were designed with the HYDEN degenerate primer design tool (16) and are listed in the Supplementary Material. The PCR was run in a touchdown fashion for 26 cycles, each cycle reducing the annealing temperature by 0.5°C starting from 68°C. A final eight cycles were performed at 55°C. All extensions were performed at 72°C. The PCR product was purified using AmpureXP and concentrated by speedvac. Dual-indexed sequencing libraries were constructed from 120 ng of PCR amplicon according to the manufacturer’s instructions of the Ovation ultralow library kit (Nugen, San Carlos, CA, USA) using custom diversity adaptors with 1–8 random nucleotides before the PCR amplicon. The library was quantified by qPCR and sequenced on a MiSeq with 2× 300 bp paired end chemistry (Illumina, San Diego, CA, USA). Material from all 10 samples was multiplexed in a single MiSeq run. Sequencing data were demultiplexed on index as well as PCR primer. Paired end reads were combined with PEAR and, subsequently, assembled using the MIGEC (17) pipeline, which processes the molecular barcode information for sequence error correction and to report expression levels without PCR bias. Default parameters were used except a minimum UMI count of 1 in MIGEC. The resulting sequences were annotated for germline alleles and regions with the High-VQuest service (18), and additional analysis was performed with the VDJTools package (19) for clustering of samples and tcR (20) to enumerate sequences overlapping between samples. VDJTools clustering was performed with the ClusterSamples function using the default distance parameter (clonotype overlap frequencies). Phylogenetic trees were built with the FastTree program and visualized with the Archaeopteryx viewer (21, 22).

Analyses on proteomics data were performed using ProgenesisQI for Proteomics 2.3 (Waters, Milford, MA, USA) for label-free quantitation and PEAKS Studio 6 (BSI Inc., Waterloo, ON, Canada) for sequence identification. Quantification is reported by ProgenesisQI as an integrated intensity under the isotopic peaks in the mass spectra, normalized for sample loading. Search parameters in PEAKS allowed for a fixed cysteine carbamidomethylation and variable methionine oxidation, a precursor mass tolerance of 10 ppm and 0.5 Da tolerance for ion trap MS–MS spectra and 1 missed cleavage. A search database was constructed from productive reads reported by High-VQuest from all samples combined. At 239 MB, the FASTA file for this database was slightly smaller in size than the common Uniprot database. Peptide spectrum matches with a −10logP confidence better than 15 were included in further analysis.

Proteomics data are made available for public use at the ProteomeXchange Consortium (23) (DOI 10.6019/PXD006484). The NGS data can be obtained from the NCBI Gene Expression Omnibus as study GSE98855 (24).

## Results

**Figure 1:**
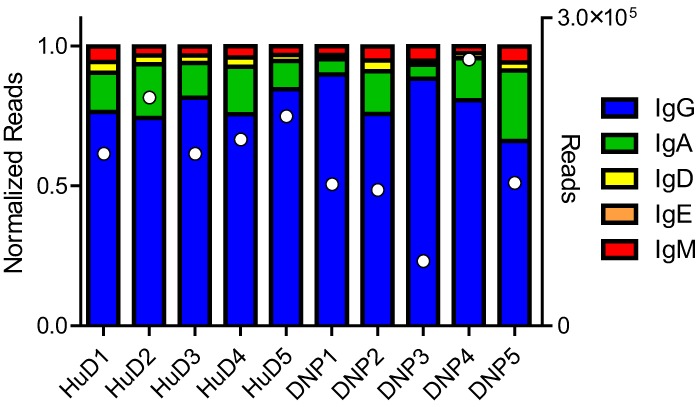
The NGS dataset included a small section of the constant domain that allows identification of the class of immunoglobulin. The reads were normalized, but the total number of processed reads included for each sample has also been plotted in white markers on the right axis. In all samples, the majority of reads belonged to the IgG class, followed by IgA and the other classes. Differences were observed between subjects but no relation to the treatment could be shown.

The immunizations and proteomics dataset were described in earlier work (10). The sequence data were demultiplexed, yielding between 1.0 × 10^6^ and 2.2 × 10^6^ paired end reads per biological sample. After processing the raw reads and collapsing those sharing the same barcodes, we assessed the class distribution of the reads. 79% of the reads related to IgG, followed by IgA, IgM, IgD, and IgE (Figure 1). As observed in other studies, the repertoire distribution was very skewed, showing a limited number of clones making up the majority of the expressed repertoire (Figure S1 in Supplementary Material).

### Sample Clustering by Antigen

**Figure 2:**
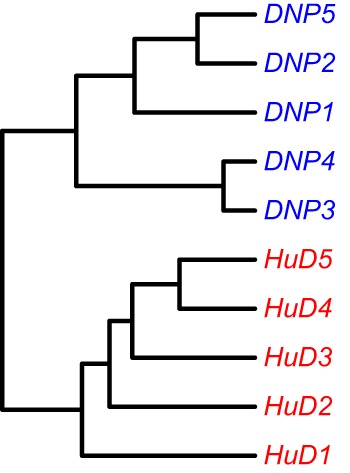
Unsupervised hierarchical clustering of samples based on NGS data on the repertoire of unique CDR3 sequences. Cluster analysis based on other regions is provided in the Supplementary Figures.

It was previously observed that animals immunized with different antigens could be distinguished from each other based on a cluster analysis on a proteomics dataset of affinity-enriched antibodies from the immune sera. A similar approach was performed on the immune repertoire data that were obtained from the splenocytes of these animals. The dataset consists of entire variable domain sequences, rather than the short peptide fragments that were identified in the proteomics data. Within the variable domain of the immunoglobulin, the complementarity-determining regions 1, 2, and 3 can be found as well as the surrounding framework regions. Unique rearrangements can be found in the CDR3 regions, and somatic mutations focus on the CDR regions but are not uncommon in the framework regions either. It is, therefore, of interest to assess several sections of the variable domain separately to assess similarities that may cluster the samples into groups. The dataset was processed with the High-VQuest service to annotate the various immunoglobulin regions in the sequence and to enable filtering for functional transcripts. The segments of interest were collected, collapsed to a unique set annotated with the read number, and the ClusterSamples function in the VDJTools package was then used to cluster the CDRs 1, 2, and 3 individually, as well as these CDRs together with their flanking framework regions. It was found that samples could be clustered according to the antigen that was used for the immunization based on all segments that were assessed, but clustering was strongest based on the CDR3 region, and became stronger by the inclusion of the flanking framework regions (Figure 2; Figure S2 in Supplementary Material).

### Correlation between Proteomics and NGS Data

Proteomics data were acquired for both total IgG and affinity-enriched IgG from all immune sera. The NGS data were derived from splenocytes taken at the same point in time from these animals. However, the splenocytes do not represent the only site of IgG production, and it was, therefore, of interest to investigate the proportion of mass spectra that could be matched to reads in the NGS dataset, as well as the correlation between the number of reads and the intensity of mass spectrometer signals for a matching spectrum.

A database was constructed from all unique immunoglobulin sequences observed in all samples combined. This database was used to match MS/MS spectra with a PEAKS DB search. Affinity-enrichment yields previously suggested that the specific IgG makes up about 0.1% of the total amount of IgG. On the other hand, the spleen may be enriched for immune cells related to an active immune response, which would be the case after immunization and boosts. As shown in Table S1 in Supplementary Material, the fraction of MS/MS spectra that could be matched to the NGS results was larger in the case of the samples of total IgG than in the case of the affinity-enriched samples. This fits with the notion that the affinity-enriched IgG is a subset of the total repertoire, and that the splenocytes can be involved in immune responses against both the immunogen and numerous other antigens. Splenocyte IgG sequences that target such other antigens will remain unmatched to proteomics data on IgG affinity-enriched for the immunogen. The pre-immune sera show an intermediate number peptide spectrum matches, indicating an overlap between serum IgG and splenocytes in spite of being sampled 3 weeks apart.

While a single unified database was used for identifications, we separately compared searches performed with a database matching or mismatching the animal used for a proteomics sample. As a mismatching database, NGS data from an animal of the alternate immunization was used. It was found that a matched proteomics-NGS dataset typically yielded more peptide spectral matches than an unmatched set, and that a matched set yielded more unique hits that were not found in the unmatched set than vice versa (Table S2 in Supplementary Material). This supports the expectation that some of the unique rearrangements in the animals can be detected by both the proteomics data and the NGS data, but still about 75% of the identifications were seen in both the matching and non-matching search. Additional searches against a UniProt database revealed that the proteomics samples consisted primarily of immunoglobulin-related peptides, but abundant serum proteins such as albumin or complement factors could be observed as well. The total number of PSMs against the rat Uniprot database was about 25% of the number found against the NGS database.

For all NGS sequences that had a match in the proteomics dataset, the number of (UMI corrected) reads containing that peptide was annotated for all samples, as well as the signal intensity in the proteomics data as determined by label-free analysis (ProgenesisQI). A good correlation between RNA and proteome data would suggest that the splenocytes are a good representation of the expressed repertoire in the serum. It was found that the correlation between affinity-enriched IgG and the splenocyte RNA was almost absent (median of pairwise correlations 0.06, Figure S3 in Supplementary Material). As above, this fits with affinity-enriched IgG as a subset of the total repertoire. The correlation of the total serum IgG with the splenocyte RNA, while still modest (median of pairwise correlations 0.24), was significantly stronger than that of the affinity-enriched data. Correlations were not increased in a subset of the data with only higher confidence PSMs (−10log*P* > 40). The incomplete correlation suggests that many of the serum antibodies were not expressed by the B-cells from the spleen or cells clonally related to them, but rather other cell populations, possibly bone marrow-resident plasma cells. However, it cannot be excluded that the incomplete repertoire coverage depth of, in particular, the proteomics data affects the correlations that were found.

### Shared Sequence Motifs

From proteomics data alone, it was previously concluded that certain peptides from antigen-specific immunoglobulins are shared among different animals, yet unique for the immunogen. In the current work, the NGS dataset presents an opportunity to validate the proteomics findings with an independent technique and to perform a more extensive exploration of the immune repertoire than was possible with the proteomics approach alone.

**Figure 3:**
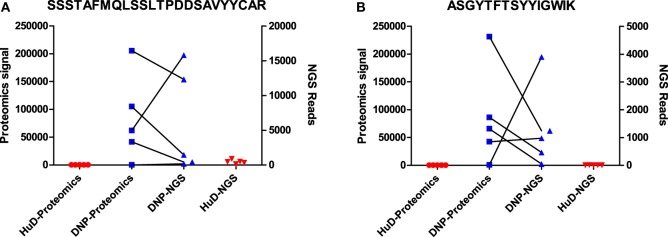
Data for two peptides [SSSTAFMQLSSLTPDDSAVYYCAR **(A)**; ASGYTFTSYYIGWIK **(B)**] that were found to be associated with DNP immune sera in proteomics data. Shown are signal intensities in proteomics, and the number of reads in NGS data containing the same peptide. Matching DNP samples have been plotted with a connecting line.

A small subset of peptides that was previously identified by proteomics as selective for one of the immunogens was evaluated in the NGS dataset. The peptide sequence was initially uncertain as only *de novo* interpretation of MS/MS spectra was available. As described in the previous section, these MS/MS spectra can be searched against a database from the matching NGS dataset, and the number of reads for matching sequences was enumerated and compared to the proteomics label-free quantitation. It was found that, in several cases, multiple unique sequences could be a potential match to the MS/MS spectra in the proteomics dataset. However, such sequences were always quite similar (leucine/isoleucine variants, or residue position swaps), and one variant always dominated the number of reads (Table S3 in Supplementary Material). The latter is most likely to correspond to the peptide that was observed in the proteomics data. Again, we found that the quantitative correlation was low, but that both datasets agree qualitatively and corroborate the observation that these peptides associate with one of the immunogens. This has been illustrated for two of these peptides in Figure 3. Some other peptides that were quite abundant and shared in proteomics data could only be matched to low numbers of reads, or did not find a match at all, showing that the overlap between the datasets is not complete.

**Figure 4:**
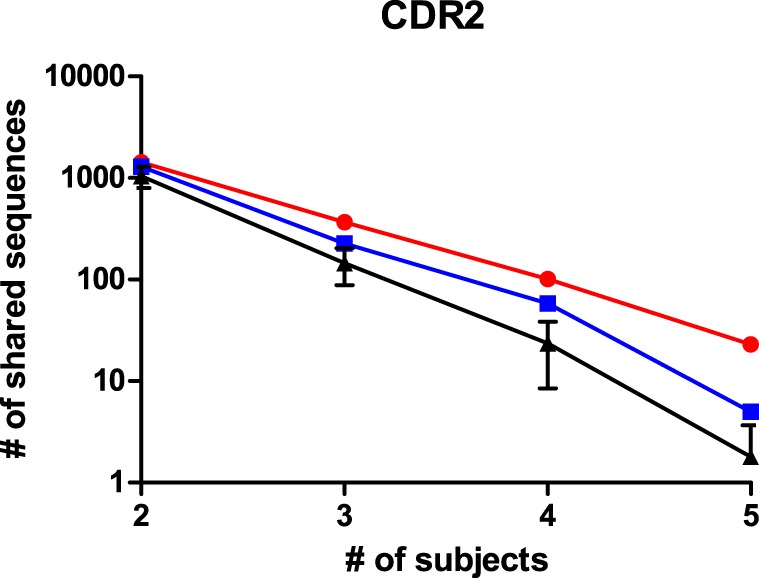
The number of shared peptides that were observed for CDR2 sequences in the NGS dataset. Peptides that were shared among animals, but still unique to one of the antigens were enumerated and shown as a function of the number of subjects among whom they were shared, for HuD immunized animals (Red), DNP-immunized animals (Blue) and randomized groups of mixed composition (black, average ± SD, *n* = 10). A similar analysis for other regions in the immunoglobulin molecule is presented in Figure S4 in Supplementary Material.

The size and read length of the NGS repertoire dataset permits a more detailed analysis of shared sequence segments. Similar to the cluster analysis, it was chosen to compare sharing for CDRs 1, 2, and 3, for CDRs expanded with the flanking framework regions, and for the complete variable domain. The datasets were processed in R with the shared.repertoire function of the tcR library (20). The output lists the number of reads that were observed in each sample for a given shared sequence segment. In these data files, it was assessed how many sequences were shared within but unique to one of the immunogen groups. This was done for both immunogen groups and also for randomized controls where sequences were to be shared between a mixed set of five animals from the dataset. For the latter, the average and SD of 10 randomized sets is shown. For all immunoglobulin segments that were tested, shared sequences were found in the datasets. The number of shared sequences was plotted against the number of animals among whom they were shared (Figure 4; Figure S4 in Supplementary Material). It was observed in all comparisons that the number of shared sequences was largest within animals immunized with the HuD antigen and less with the DNP antigen. In the scrambled control sets, sharing was less than within either HuD- or DNP-treated animals, which were typically outside the 95% confidence limit of these controls. The presence of shared sequence segments within a mixed control group could indicate some sharing events occur by chance alone, although all immunized animals were also littermates that did share exposure to other environmental factors. Therefore, all subjects can share immune responses against antigens other than the intended immunogen as well. Figure 4 and Figure S4 in Supplementary Material showed the extent of sharing for peptides that are found in animals treated with one antigen but that are not found for the alternate antigen. The analysis was repeated for shared peptides but without further constraint on presence or absence in animals with the alternate antigen, and the results for the CDR3 were also included in a panel of Figure S4 in Supplementary Material. Without the constraint more shared peptides were found, but differences between antigens and random controls were reduced. Similar results were found for the other regions of the immunoglobulin molecule. Although the absolute number of shared sequences varied depending on the region of the immunoglobulin molecule, in all cases the number of shared sequences decreased in a log-linear fashion as a function of the number of samples in which they were shared. If this trend also holds for a larger number of subjects than studied here, this implies that the sharing of any single sequence segment among all members of a large population that responded to an immunogen will be quite rare. This may affect the design of diagnostic applications that rely on shared motifs in the immune repertoire.

For the CDRs 1 and 2, the number of shared sequences is increased when the flanking framework regions are included in the analysis. It may seem counterintuitive that longer sequences are shared more, but this could be explained by CDR sequences that are split up into unique entities because of distinct motifs in their framework regions and that are, thus, counted multiple times versus only once when considering the CDR alone. This observation does not hold for the CDR3, which shows similar levels of sharing with and without the surrounding frameworks. Possibly this relates to the higher diversity in de CDR3 and the lower number of reads per unique CDR3.

We analyzed the size distribution of the CDR3 sequences that were shared among animals and compared them to the size distribution for the CDR3 in the entire dataset. We found that the shared CDR3 sequences were significantly shorter, which is consistent with other reports in the literature (13) (Figure S5 in Supplementary Material). While very long CDR3s have been associated with long-lasting and well-developed immune responses, it has also been shown that shared and shorter CDR3 sequences still encode for antigen-specific sequences (13, 25). While the sharing of shorter CDR3 sequences seems to be favored; it is, therefore, still expected that the shared sequences represent normal antigen-specific antibodies.

### Phylogenetic Analysis

**Figure 5:**
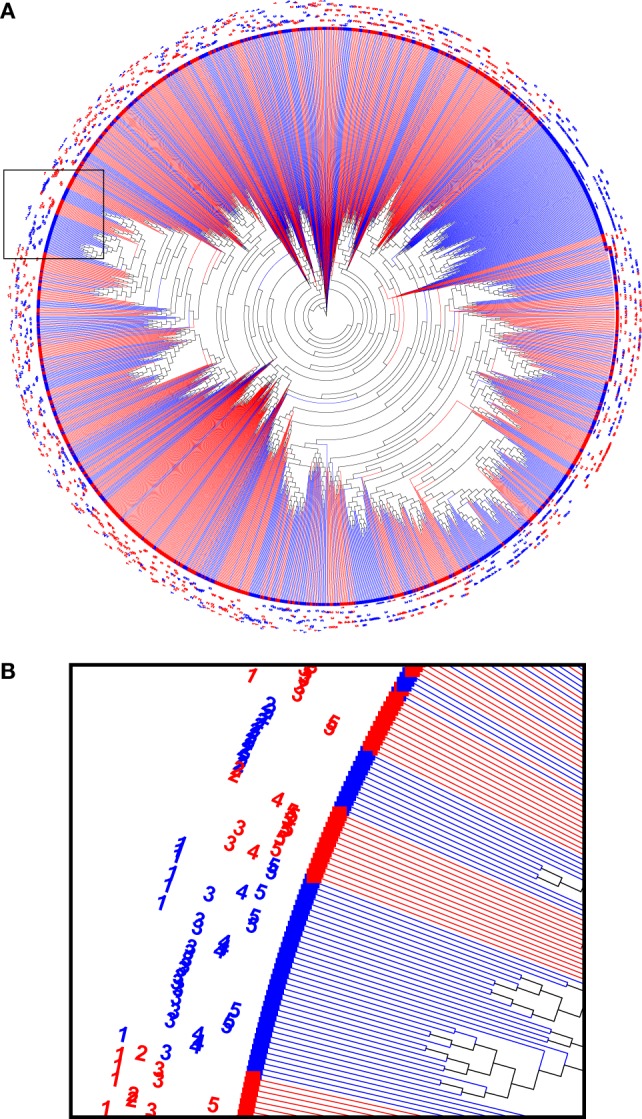
A phylogenetic tree computed from a combined set of the 200 most abundant reads from each sample **(A)**. Nodes from HuD were plotted in red and nodes from DNP-treated animals were plotted in blue, and sample identifiers (1–5) were included in the label. Branches that were homogeneous for the antigen that was used (single color) often contained reads derived from all of the individual animals of that group. The indicated region that was enlarged **(B)** highlights examples of both mixed sample branches and a single sample (DNP-2) branch.

In order to interpret the relations between the immunoglobulin sequences in the sample, a phylogenetic analysis was performed. First, the 200 most abundant reads were taken from each biological sample, combined and aligned as IMGT-gapped amino acid sequences 1–108 and processed with FastTree. The resulting data were visualized as a circular phylogram with the Archaeopteryx viewer and color coded for the treatment group (Figure 5). Several branches can be identified in the phylogram that contained reads from only one treatment group, but that still represented all animals from that group. This suggests that the immunization led to homologous sets of sequences, also among the more highly expressed sequences.

**Figure 6:**
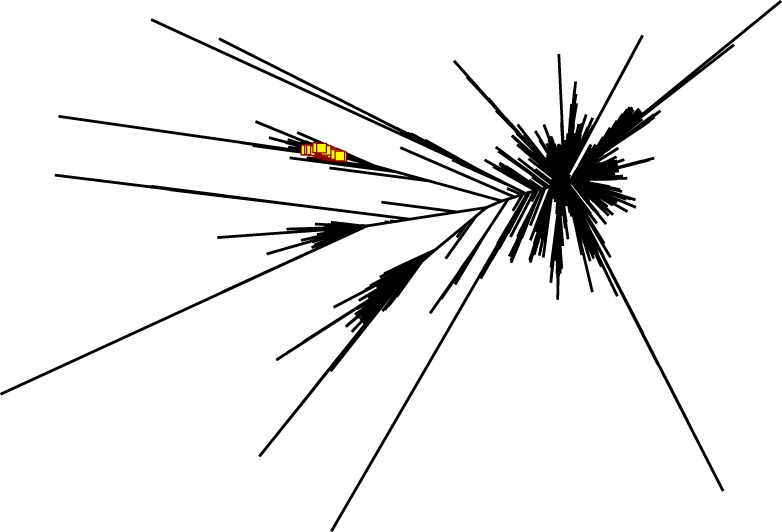
Phylogram generated from productive reads of one of the DNP-immunized animals. The peptide ASGYTFTSYYIGWIK was abundantly found in affinity-enriched immunoglobulins in the proteomics data. NGS reads that contain peptide motif were highlighted in red in the phylogram, and show a set of DNP-specific reads in relation to the overall repertoire. Figures for the other animals in this group are in Figure S7 in Supplementary Material.

**Figure 7:**
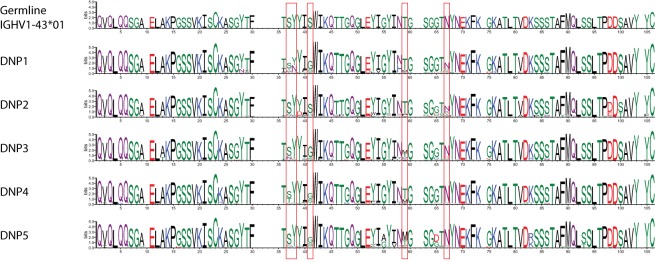
Weblogo plots of the sequences contained in the phylogram branch encompassing all nodes highlighted in red in Figure S7 in Supplementary Material. A consensus sequence was extracted from the plots, and the most homologous germline sequence was determined with the DomainGapAlign function provided by IMGT. The sequence of this was included as a similar weblogo in the Figure as a reference.

While such sequences probably relate to the antigens of interest, we further explored phylogenetic relations based on a peptide (ASGYTFTSYYIGWIK) that emerged from the proteomics data of affinity-enriched anti-DNP antibodies. While this peptide was also found among the high abundant subset analyzed above, we computed phylogenetic trees based on all productive reads from the MIGEC/High-V-Quest processing but for each animal separately. Subsequently, all reads containing the peptide were highlighted in an unrooted tree diagram (Figure 6; Figure S6 in Supplementary Material). These reads, probably related to anti-DNP immunoglobulins, clustered in distinct branches in the total repertoire. Subsequently, sequences from all nodes of such branches were listed, and for each sample a weblogo plot was constructed of the sequence repertoire, as well as one for the most homologous germline sequence (26). In these diagrams, it can be observed that, within the consensus sequence, up to four residue mutations are favored at selected positions, while otherwise the repertoire does not deviate much from the germline sequence (Figure 7).

### Proteogenomic Analysis of Repertoires

The availability of NGS data enables an extended analysis of the proteomics data, and also an analysis of some discrepancies that were observed in the past. One limitation of bottom-up proteomics is the limited sequence length covered by a tryptic peptide. The large set of homologous peptides in an immunoglobulin digest makes is impossible to reconstruct the full protein sequence associated with a given peptide of interest. By matching the peptide MS/MS spectrum to the NGS dataset, full variable region sequences can be identified that contain the peptide of interest. Moreover, these full length sequences may contain other tryptic peptides that are also represented in the proteomics dataset. Thus, observations from one peptide may now be supported by observations on additional peptides from the same chain, which could otherwise not be recognized as related. We found several examples of such peptides, and indeed such peptides showed similarities in their abundance in the samples (Figure S7 in Supplementary Material). Still, differences were observed as well, which may relate to the fact that, while peptides occur within the same chain for a subset of the reads, also other reads are present within the repertoire that contain either one peptide or the other but not both.

**Figure 8:**
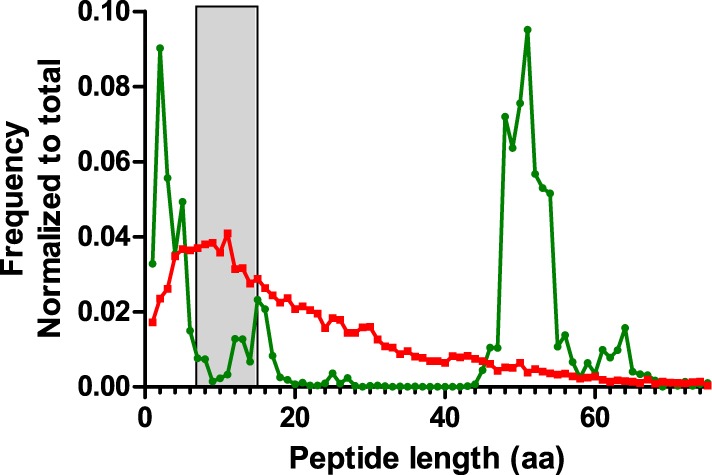
Length distribution of tryptic peptides encompassing the CDR3 region obtained by *in silico* digestion of reads combined from all samples (green). The NGS data were appended with rat IgG2B sequence in order to include the first tryptic site located in the CH1 domain. For comparison, a similar digestion was performed on the human subset of the Uniprot protein database (red). The peptide size range that is optimal for shotgun proteomics analysis (7–15aa) has been highlighted in gray.

During the analysis of the proteomics dataset, it was observed that CDR3 sequences were underrepresented in the results. It was unclear whether this related to limitations in sample preparation, detection in the instrument, or proper identification and recognition of these polymorphic regions as CDR3. One cause for poor detection can be long peptide lengths. For proteomics, the optimal peptide lengths range between 7 and 15 amino acids. We performed an *in silico* digestion of the NGS dataset using the Bio:Protease Perl package, and enumerated the length of tryptic peptides encompassing the CDR3, defined as a peptide with tryptic sites that surround IMGT-numbered residue 107 (Figure 8). The analysis revealed that the distribution peaked around 50-aa length, which is a length that adversely affects peptide detection. A secondary peak was observed for peptides of 2–5 aa, which is rather too short for detection and specificity. For comparison, the same algorithm was used to process a database with the human subset of the Uniprot protein database. That distribution, although long-tailed, peaks within the optimal range of 7–15 amino acids and is clearly different from CDR3 peptides. The data show that the amino acid composition of the CDR3 region is poorly suited for conventional trypsin-based proteomics and would benefit from either alternative proteases or from instrumental capabilities for larger molecules, such as high mass resolution and ETD fragmentation rather than only CID.

## Discussion

The data presented combine NGS and proteomics analysis to show that immune responses result in antibody sequence fragments that are shared among subjects exposed to the same immunogen. The new NGS data provide a much deeper view on the immune repertoire, as well as an improved sequence accuracy. The proteomics data, however, still allows us to focus on an antigen-specific subset of immunoglobulin sequences.

### Similarity

A cluster analysis of the NGS data confirmed that, indeed, the immune repertoire of animals exposed to the same antigen contains similarities that allow them to be grouped accordingly. While this agrees with a similar analysis on proteomics data, this finding is nevertheless remarkable. First, the NGS data are based on the entire splenocyte repertoire and not on an antigen-specific subset of it. The majority of the repertoire is expected to relate to antigens other than the immunogen, but as the animals were treated the same except for the immunogen the antigen-specific response is the most likely component that drives the clustering. Second, it was found that sample clustering could be based on all complementarity-determining regions in the immunoglobulin molecule, including the CDR3. The latter was poorly represented in the proteomics dataset, and is considered the most diverse region of the molecule, driven by the random recombination of V, D, and J segments. Still, clustering of samples based on CDR3 sequences followed similar patterns as for the other CDRs, showing that homology and convergence occur in all of them.

### Shared Motifs

The repertoires of the samples were not only similar, many sequences could be identified that were exact matches between several animals in the dataset. The sharing of sequences in adaptive immune responses has been controversial in literature. While sharing has frequently been observed, interpretation whether this is a stochastic event driven by random chance, an experimental artifact or a selective process that demonstrates convergence in the immune repertoire remains under debate (11–13, 27–29). We looked into the NGS data for evidence of index hopping, an artifact that might explain shared sequences. However, we did not find reads with the same sequence and UMI barcode in different samples, indicating that the dual-index library preparation successfully prevented this problem. From the current dataset, we conclude that both chance and selection contribute to the sharing of sequences. Observations that support stochastic contributions are the decrease in the numbers of shared sequences as a function of the number of animals in which it is observed, the presence of shared sequences among animals treated with different immunogens, and shorter size distribution of CDR3 sequences in shared subsets compared to the total distribution of CDR3 sizes. On the other hand, the data show that sequence sharing is consistently higher among samples that share the same immunogen, and that samples can be properly grouped based on sequence similarities in their immune repertoires. Such observations support the view that repertoire convergence does indeed occur for the antigens that were investigated in this work.

Studies in the literature typically report convergence and sharing of the CDR3 region of the heavy chain, as this is known as the most diverse and most decisive for antigen specificity (13, 30). Here, we extend the analysis to the entire length of the variable domain and find that shared motifs can also be identified in and around the other two CDRs in the molecule. While the CDR3 may be the primary determinant of immunoglobulin specificity, finding that CDRs 1 and 2 similarly converge in response to an antigen indicates that they are still subject to somatic mutations and selection pressures and, thus, have a considerable contribution to binding (31).

### Antigen Dependence

Repertoire convergence has been reported for a limited but increasing number of antigens and conditions, including HIV, tetanus toxoid, *Streptococcus pneumoniae, Haemophilus influenzae*, Dengue fever and Sjögren’s syndrome (9, 11, 12, 32, 33). We now add evidence for the HuD antigen and DNP epitopes and have done so with two independent techniques. The question arises whether such sharing can be expected from a majority of antigens, or that it should be considered an exception. The only definitive answer to this question can be provided by collecting a large collection of repertoire data for different individuals and antigens. While such comprehensive data are still lacking, the number of datasets showing sharing or convergence is increasing while datasets that demonstrate absence thereof remain lacking. We, therefore, expect the sharing of motifs in the immune repertoire to be a more general phenomenon. We did find differences between both antigens in terms of the degree of sequence sharing between the repertoires, with the HuD antigen consistently sharing more sequence motifs than the DNP antigen. The HuD antigen is bigger and can expose more epitopes than the small DNP group. However, this should not have a big effect, as the NGS data consider not a specific subset but all antibodies in the repertoire, which may also include those targeting the KLH carrier protein used for DNP. Potential artifacts that could explain our observations were investigated, but no evidence was found for problems in index cross-contamination, or for significant differences in the number of reads for the samples. It is, therefore, more plausible that antigens differ intrinsically in the amount of sequence sharing that they elicit. It is conceivable that the fraction of distinct VDJ rearrangements that leads to specific binding early in the B-cell response inversely correlates with the degree of sharing between individuals, but a more extensive dataset covering more than two antigens is required to support that concept with evidence.

### Immuno Proteogenomics

The combined use of proteomics and NGS was intended to obtain complementary information on the state of the immune repertoire. Indeed, it was possible to find evidence for repertoire convergence in both datasets, and specific observations could be cross-validated. On the other hand, the degree of quantitative correlation between the datasets was limited, and the fraction of MS–MS spectra that could be matched to NGS reads was relatively low. This suggests that the repertoires of expressed serum IgG and splenocyte mRNA overlap, but only partially. It has been reported that proteomics identification of immunoglobulins is more challenging than that of other proteomes (34). Also, as shown in Figure 8, workflows using trypsin have challenges for proteomics data that cover the CDR3. While specific peptide size distributions may shift for other immunoglobulin classes due to their CH1 sequence and also for other species, we expect that the length of CDR3 tryptic peptides can be a common problem. We did not observe an improvement of correlation between proteomics and NGS in a subset of high scoring PSMs from the dataset, and we, therefore, do not believe that misidentification plays an important role in the discrepancies that are observed. Rather, we suspect the expression of serum immunoglobulins in niches that were not covered by RNA sequencing, such as bone marrow plays an important role, as well as the sequencing of RNA of other cell lineages that does not encode immunoglobulins, but rather the B-cell receptor. Refinement of experimental choices and protocols should bring NGS and proteomics data closer into alignment, and indeed several successful datasets have been described in the literature (7, 8, 35, 36).

### Potential Applications

While it is clear that many sequences are shared among animals exposed to the same antigen, the data also show that the degree of sharing drops in a log-linear fashion as a function of the number of sharing individuals. This implies that it is unlikely that any one sequence motif can function as a marker for a large population of subjects. If this would be possible, a simple (proteomics) assay for the presence of such an amino acid motif could function as a proxy for a variety of immunological conditions, such as response to a vaccination (37), pathogen infection, auto-immune disease, or a response against tumor-associated antigens. While such an application based on a single peptide seems unlikely based on the current dataset, it is still conceivable that a panel of peptides or peptide homology to a conserved motif could fulfill such a purpose. A test based on composite markers would be more complex, and proper discovery and validation of such markers would require more extensive datasets that protect against false discovery and give sufficient confidence that a candidate motif indeed is predictive of an immune response in a larger population.

Other applications may include the identification of specific clonal expansions in immune sera for the production of monoclonal antibodies, or characterization of immune responses that target pathogens or in auto-immune conditions. In recent work, it has been shown that in the T-cell receptor, sequences are not only shared between individuals, but it is even feasible to predict epitope specificity based on new unseen sequence data (38, 39). Extension of such methods to immunoglobulin repertoires could accelerate the development of new applications of repertoire analysis.

An important aspect of future work is the location of sampling for repertoire analysis. Lymphoid organs and lymphocytes infiltrating at disease locations are very much of interest, but may be unrealistic sampling locations for many human applications. A comparison of such sampling locations with more readily accessible PBMCs, also in relation to time after antigen exposure, could help decision making on the best strategy in the development of new applications. Such an analysis could also include a comparison on which combination of cellular compartments best reflects immunoglobulin proteins that can be observed in serum, and which combination of compartments is most representative for the immunology of localized disease processes in an organism. It is also conceivable that the sharing of sequences that we observe in spleen and serum is more pronounced when considering a distinct niche, for example local to the disease. While modern NGS and proteomics techniques provide rapidly expanding views on the makeup of the adaptive immune response, the underlying processes and dynamics remain incompletely understood.

## Author Contributions

MV contributed to conception, NGS amplicon generation, proteomics sample preparation, data analysis, and drafting of the manuscript; LD contributed to conception and proteomics data acquisition; WI developed the customized library preparation and was responsible for NGS data acquisition; PS-S contributed to conception of the work; TL contributed to conception and drafting of the manuscript. All the authors critically read, contributed, and approved the manuscript.

## Conflict of Interest Statement

The authors declare that the research was conducted in the absence of any commercial or financial relationships that could be construed as a potential conflict of interest. The reviewer AH and handling editor declared their shared affiliation.

## References

[1] Tonegawa S Somatic generation of antibody diversity. Nature (1983) 302(5909):575–81. 10.1038/302575a0 6300689

[2] Friedensohn S Khan TA Reddy ST. Advanced methodologies in high-throughput sequencing of immune repertoires. Trends Biotechnol (2017) 35(3):203–14. 10.1016/j.tibtech.2016.09.010 28341036

[3] Galson JD Clutterbuck EA Truck J Ramasamy MN Munz M Fowler A BCR repertoire sequencing: different patterns of B-cell activation after two meningococcal vaccines. Immunol Cell Biol (2015) 93(10):885–95. 10.1038/icb.2015.57 25976772 PMC4551417

[4] Obermeier B Mentele R Malotka J Kellermann J Kümpfel T Wekerle H Matching of oligoclonal immunoglobulin transcriptomes and proteomes of cerebrospinal fluid in multiple sclerosis. Nat Med (2008) 14(6):688–93. 10.1038/nm1714 18488038

[5] Georgiou G Ippolito GC Beausang J Busse CE Wardemann H Quake SR The promise and challenge of high-throughput sequencing of the antibody repertoire. Nat Biotechnol (2014) 32(2):158–68. 10.1038/nbt.2782 24441474 PMC4113560

[6] Lavinder JJ Horton AP Georgiou G Ippolito GC. Next-generation sequencing and protein mass spectrometry for the comprehensive analysis of human cellular and serum antibody repertoires. Curr Opin Chem Biol (2015) 24:112–20. 10.1016/j.cbpa.2014.11.007 25461729

[7] Ogishi M Yotsuyanagi H Moriya K Koike K. Delineation of autoantibody repertoire through differential proteogenomics in hepatitis C virus-induced cryoglobulinemia. Sci Rep (2016) 6:29532. 10.1038/srep29532 27403724 PMC4941579

[8] Chen J Zheng Q Hammers CM Ellebrecht CT Mukherjee EM Tang HY Proteomic analysis of pemphigus autoantibodies indicates a larger, more diverse, and more dynamic repertoire than determined by B cell genetics. Cell Rep (2017) 18(1):237–47. 10.1016/j.celrep.2016.12.013 28052253 PMC5221611

[9] Zhou J Lottenbach KR Barenkamp SJ Lucas AH Reason DC. Recurrent variable region gene usage and somatic mutation in the human antibody response to the capsular polysaccharide of *Streptococcus pneumoniae* type 23F. Infect Immun (2002) 70(8):4083–91. 10.1128/IAI.70.8.4083-4091.2002 12117915 PMC128163

[10] VanDuijn MM Dekker LJ Zeneyedpour L Smitt PA Luider TM. Immune responses are characterized by specific shared immunoglobulin peptides that can be detected by proteomic techniques. J Biol Chem (2010) 285(38):29247–53. 10.1074/jbc.M110.139071 20615873 PMC2937956

[11] Arentz G Thurgood LA Lindop R Chataway TK Gordon TP. Secreted human Ro52 autoantibody proteomes express a restricted set of public clonotypes. J Autoimmun (2012) 39(4):466–70. 10.1016/j.jaut.2012.07.003 22871259

[12] Parameswaran P Liu Y Roskin KM Jackson KK Dixit VP Lee JY Convergent antibody signatures in human dengue. Cell Host Microbe (2013) 13(6):691–700. 10.1016/j.chom.2013.05.008 23768493 PMC4136508

[13] Truck J Ramasamy MN Galson JD Rance R Parkhill J Lunter G Identification of antigen-specific B cell receptor sequences using public repertoire analysis. J Immunol (2015) 194(1):252–61. 10.4049/jimmunol.1401405 25392534 PMC4272858

[14] Wang C Liu Y Cavanagh MM Le Saux S Qi Q Roskin KM B-cell repertoire responses to varicella-zoster vaccination in human identical twins. Proc Natl Acad Sci U S A (2015) 112(2):500–5. 10.1073/pnas.1415875112 25535378 PMC4299233

[15] Leypoldt F Wandinger KP. Paraneoplastic neurological syndromes. Clin Exp Immunol (2014) 175(3):336–48. 10.1111/cei.12185 23937626 PMC3927895

[16] Linhart C Shamir R. The degenerate primer design problem. Bioinformatics (2002) 18(Suppl 1):S172–81. 10.1093/bioinformatics/18.suppl_1.S172 12169545

[17] Shugay M Britanova OV Merzlyak EM Turchaninova MA Mamedov IZ Tuganbaev TR Towards error-free profiling of immune repertoires. Nat Methods (2014) 11(6):653–5. 10.1038/nmeth.2960 24793455

[18] Brochet X Lefranc MP Giudicelli V. IMGT/V-QUEST: the highly customized and integrated system for IG and TR standardized V-J and V-D-J sequence analysis. Nucleic Acids Res (2008) 36(Web Server issue):W503–8. 10.1093/nar/gkn316 18503082 PMC2447746

[19] Shugay M Bagaev DV Turchaninova MA Bolotin DA Britanova OV Putintseva EV VDJtools: unifying post-analysis of T cell receptor repertoires. PLoS Comput Biol (2015) 11(11):e1004503. 10.1371/journal.pcbi.1004503 26606115 PMC4659587

[20] Nazarov VI Pogorelyy MV Komech EA Zvyagin IV Bolotin DA Shugay M tcR: an R package for T cell receptor repertoire advanced data analysis. BMC Bioinformatics (2015) 16:175. 10.1186/s12859-015-0613-1 26017500 PMC4445501

[21] Han MV Zmasek CM. phyloXML: XML for evolutionary biology and comparative genomics. BMC Bioinformatics (2009) 10:356. 10.1186/1471-2105-10-356 19860910 PMC2774328

[22] Price MN Dehal PS Arkin AP FastTree 2 – approximately maximum-likelihood trees for large alignments. PLoS One (2010) 5(3):e9490 10.1371/journal.pone.0009490 20224823 PMC2835736

[23] Vizcaino JA Deutsch EW Wang R Csordas A Reisinger F Rios D ProteomeXchange provides globally coordinated proteomics data submission and dissemination. Nat Biotechnol (2014) 32(3):223–6. 10.1038/nbt.2839 24727771 PMC3986813

[24] Edgar R Domrachev M Lash AE. Gene expression omnibus: NCBI gene expression and hybridization array data repository. Nucleic Acids Res (2002) 30(1):207–10. 10.1093/nar/30.1.207 11752295 PMC99122

[25] Yu L Guan Y. Immunologic basis for long HCDR3s in broadly neutralizing antibodies against HIV-1. Front Immunol (2014) 5:250. 10.3389/fimmu.2014.00250 24917864 PMC4040451

[26] Crooks GE Hon G Chandonia JM Brenner SE. WebLogo: a sequence logo generator. Genome Res (2004) 14(6):1188–90. 10.1101/gr.849004 15173120 PMC419797

[27] Weinstein JA Jiang N White RA III Fisher DS Quake SR High-throughput sequencing of the zebrafish antibody repertoire. Science (2009) 324(5928):807–10. 10.1126/science.1170020 19423829 PMC3086368

[28] Vollmers C Sit RV Weinstein JA Dekker CL Quake SR. Genetic measurement of memory B-cell recall using antibody repertoire sequencing. Proc Natl Acad Sci U S A (2013) 110(33):13463–8. 10.1073/pnas.1312146110 23898164 PMC3746854

[29] Hoehn KB Fowler A Lunter G Pybus OG. The diversity and molecular evolution of B-cell receptors during infection. Mol Biol Evol (2016) 33(5):1147–57. 10.1093/molbev/msw015 26802217 PMC4839220

[30] Xu JL Davis MM. Diversity in the CDR3 region of V(H) is sufficient for most antibody specificities. Immunity (2000) 13(1):37–45. 10.1016/S1074-7613(00)00006-6 10933393

[31] Chen C Roberts VA Rittenberg MB. Generation and analysis of random point mutations in an antibody CDR2 sequence: many mutated antibodies lose their ability to bind antigen. J Exp Med (1992) 176(3):855–66. 10.1084/jem.176.3.855 1512548 PMC2119366

[32] Poulsen TR Meijer PJ Jensen A Nielsen LS Andersen PS. Kinetic, affinity, and diversity limits of human polyclonal antibody responses against tetanus toxoid. J Immunol (2007) 179(6):3841–50. 10.4049/jimmunol.179.6.3841 17785821

[33] Gorny MK Wang XH Williams C Volsky B Revesz K Witover B Preferential use of the VH5-51 gene segment by the human immune response to code for antibodies against the V3 domain of HIV-1. Mol Immunol (2009) 46(5):917–26. 10.1016/j.molimm.2008.09.005 18952295 PMC2693011

[34] Boutz DR Horton AP Wine Y Lavinder JJ Georgiou G Marcotte EM. Proteomic identification of monoclonal antibodies from serum. Anal Chem (2014) 86(10):4758–66. 10.1021/ac4037679 24684310 PMC4033631

[35] Sato S Beausoleil SA Popova L Beaudet JG Ramenani RK Zhang X Proteomics-directed cloning of circulating antiviral human monoclonal antibodies. Nat Biotechnol (2012) 30(11):1039–43. 10.1038/nbt.2406 23138294

[36] Lavinder JJ Wine Y Giesecke C Ippolito GC Horton AP Lungu OI Identification and characterization of the constituent human serum antibodies elicited by vaccination. Proc Natl Acad Sci U S A (2014) 111(6):2259–64. 10.1073/pnas.1317793111 24469811 PMC3926051

[37] Galson JD Pollard AJ Truck J Kelly DF. Studying the antibody repertoire after vaccination: practical applications. Trends Immunol (2014) 35(7):319–31. 10.1016/j.it.2014.04.005 24856924

[38] Dash P Fiore-Gartland AJ Hertz T Wang GC Sharma S Souquette A Quantifiable predictive features define epitope-specific T cell receptor repertoires. Nature (2017) 547(7661):89–93. 10.1038/nature22383 28636592 PMC5616171

[39] Glanville J Huang H Nau A Hatton O Wagar LE Rubelt F Identifying specificity groups in the T cell receptor repertoire. Nature (2017) 547(7661):94–98. 10.1038/nature22976 28636589 PMC5794212

