# Supplementary material for: Immune Repertoire after Immunization As Seen by Next-Generation Sequencing and Proteomics
Source: Front Immunol. 2017 Oct 16;8:1286. doi: 10.3389/fimmu.2017.01286 (PMC5650670; doi:10.3389/fimmu.2017.01286)
Supplement: Supplementary file 1 [file Presentation_1.PDF]

frontiers

Supplementary Material

Immune repertoire after immunization as seen by next generation sequencing and proteomics

Martijn M. VanDuijn*, Lennard J. Dekker, Wilfred FJ van IJcken, Peter A.E. Sillevis Smitt and Theo M. Luider

*** Correspondence:** Corresponding Author: m.m.vanduijn@erasmusmc.nl

1 Supplementary Methods

A mixture of the following primers were used for reverse transcription of mRNA into cDNA. N represents a random nucleotide that is used as an UMI tag. As the UMI tag was later found to be of insufficient length, a 10-base section from the CDR3 was as used as a complimentary UMI tag during data analysis. A T7 motif (TAATACGACTCACTATAGGGAG) is introduced to the cDNA for later use as a common priming site during PCR.

```
>RatIGHG_Rev_UID_T7
TAATACGACTCACTATAGGGAGNNNNNNAGACAGATGGGGCTGTTGTT
>RatIGHA_Rev_UID_T7
TAATACGACTCACTATAGGGAGNNNNNNGGGGGTCTCAGTGGGTAGAT
>RatIGHD_Rev_UID_T7
TAATACGACTCACTATAGGGAGNNNNNNCATTTTGATTTGGGGCTTTG
>RatIGHE_Rev_UID_T7
TAATACGACTCACTATAGGGAGNNNNNNCAGGGCTTCAAGGGATAGAG
>RatIGHM_Rev_UID_T7
TAATACGACTCACTATAGGGAGNNNNNNGGCCACCAAATTCTCATCA
```

For PCR, the common T7 reverse primer was used, and a mixture of degenerate primers complementary to the Leader segment of the variable genes as forward primers. These primers were derived from the rat germline repertoire with the HYDEN software tool. The primers used for PCR have been as listed below (IUPAC notation).

```
>T7
TAATACGACTCACTATAGGGAG

>RatL_V01_1.24b
TGGARCTKGRTCTTTCTCTTYYTC
>RatL_V01_2.24b
CATYCTCTTCTWGATGGMASTARC
>RatL_V01_3.24b
GGTGTYYACTCYCAGRTYCAGCTG
>RatL_V03_1.24b
TSCTCTRCCTGRTGACHTTTCCAA
>RatL_V03_2.24b
TGRCTRTCCTGGTGCTKYTSCTCT
>RatL_V03_3.24b
CCTSTACCTGGTGMCTTTTMSARG
>RatL_V03_.24b
CTTCTGTACCTGTTGACAGCCCTT
>RatL_V04_.24b
TAAAAGGTGTCCAGTGTGAGGTGA
>RatL_V05_2.24b
GTTTTCYTTKTBYTTATTTTAAAA
>RatL_V05_3.24b
TTGKTCTCAGWTGHTATRGATGCC
>RatL_V06_1.24b
TTYCTTGTTGYWCTTTTVAAAGGT
>RatL_V06_2.24b
TTRAAAGGYGYCCWRTGTGAGGTG
>RatL_V07_.24b
TAACACTTTTAAATGGDATYSRGT
>RatL_V08_.24b
CTTACYTYCTCATTCCTKCTGCTG
>RatL_V09_.24b
AAAGTGCYCAAGCAYAGATWCAGT
>RatL_V10_1.24b
TDTGCAYTGTGAGGTRCAGSTTGT
>RatL_V10_2.24b
TTTYTTTTAARGGGAYACATTGYR
>RatL_V11_.24b
AWTTTTTATTTTTGYTCTYTTDAA
>RatL_V12_.24b
CTGTGCTTGGCAGCMAYTCTGCAA
>RatL_V15_.24b
TTGGATTATGCTTTATCTGCTGGC
```

2 Supplementary Figure Captions

Supplementary Figure 1.

A limited number of clones dominates the repertoire. For each subject, unique CDR3 sequences were ranked by the number of reads in which they were observed. The 1000 most abundant clones were plotted against the number of reads for each sample. On average, the 50 most abundant sequences made up 40% of the reads. DNP immunized animals were plotted in blue, and HuD immunized in red. Individual animals 1-5 were marked with circles, triangles, inverted triangles, squares and diamonds, respectively.

Supplementary Figure 2

Unsupervised hierarchical clustering of samples based on NGS repertoire data on unique sequences in the dataset. The analysis was performed for CDR1, CDR2 and CDR3, as well as these CDRs with their surrounding framework regions, and also based on the entire variable domain (Framework1 until Framework4.

Supplementary Figure 3

For peptides that could be matched between proteomics and NGS datasets, the number of reads in the NGS data was plotted against the signal intensity in the proteomics data. 0 was plotted as 0.1 on the log axis, and a random jitter (0.05) was applied to reduce overlapping datapoints. This analysis is shown for proteomics data from total serum IgG (A; median pairwise correlation 0.24) and for affinity enriched IgG (B; median pairwise correlation 0.06). The data shown is for all samples combined, qualitatively similar plots were obtained for comparisons between individual samples.

Supplementary Figure 4

The number of shared peptides that was observed for regions in the NGS dataset. Peptides that were shared among animals, but still unique to one of the antigens were enumerated and shown as a function of the number of subjects among whom they were shared, for HuD immunized animals (Red), DNP immunized animals (Blue) and randomized groups of mixed compositions (black, average ± SD, n=10). The region of the immunoglobulin molecule that was analyzed is indicated above each plot. In the plot labelled “CDR3 - Not Antigen specific”, shared peptides were plotted without considering presence or absence of the peptide in animals that were immunized with the alternate antigen.

Supplementary Figure 5

Shared CDR3 sequences (green circles) consist of a shorter subset of the total set of CDR3 sequences (black squares) in the NGS dataset (t-test $p<2.2 \times 10^{-16}$). The distribution of CDR3 lengths in the plot was normalized to the total number of sequences in each of the sets. Total CDR3 sequences were collected from all animals, and for the shared subset those common to two or more animals were selected. No additional length effect could be seen in subsets shared among larger numbers of subjects.

Supplementary Figure 6

Unrooted phylograms generated from productive reads of each of the DNP immunized animals. The peptide ASGYTFTSYYIGWIK was abundantly found in affinity enriched immunoglobulins in the proteomics data. NGS reads that contain that peptide motif were highlighted in red in the phylogram, and show some of the DNP-specific reads in relation to the overall repertoire.

Supplementary Figure 7

Signal intensities in proteomics data for two peptides that were found to co-occur in reads in the NGS dataset. Signal intensities can only provide a relative comparison without additional calibrators. Signal intensities below 10000 often suffer from poor signal to noise ratios in this type of label-free analysis. A representative read from the dataset (subject HuD1) shows both peptides in the context of the variable domain.

Supplementary Table 1

Summary of the proteomics data. Shown are data from immune sera, both total IgG and affinity enriched IgG, as well as IgG from pre-immune sera. For each animal in the dataset, the total number of MS/MS spectra recorded , the spectra therein that could be matched to a sequence in the NGS data (PSM -logP>15), and the number of unique peptide sequences within those matches have been noted in the table.

Supplementary Table 2

Proteomics results for matching or non-mathcing databases. For the animals indicated in the rows, a search was performed with PEAKS DB against NGS data obtained from the same animal (‘Matched’), or NGS data from another animal (‘Unmatched’). For an unmatched search, the corresponding animal immunized with the alternate antigen was used, e.g. animal DNP-1 database for animal HuD-1 proteomics. For every search, the number of peptide spectral matches is shown, the number of peptide spectral matches that were found for both searches, and the peptide spectral matches that were uniquely found in that search, and not in the search with the alternate database.

Supplementary Table 3

Matching of proteomics data to the NGS sequence database. Shown are two peptides with their associated mass to charge ratio. The MS/MS data from these peptides could be matched to distinct sequences within the NGS data that are all shown. For each of these peptides, the number of reads containing them has been plotted for each sample. In the proteomics data, both peptides were elevated in DNP immune sera.

A

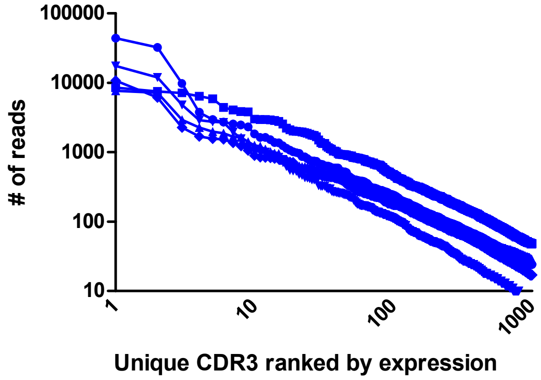

B

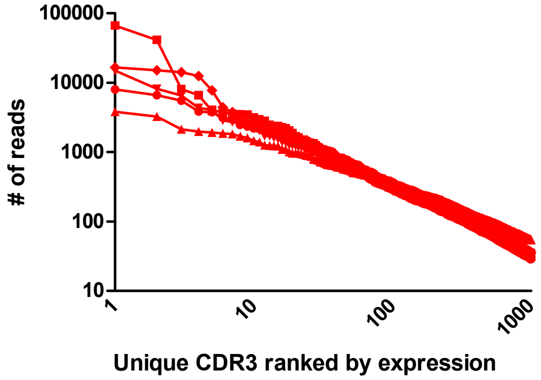

Supplementary Figure 1

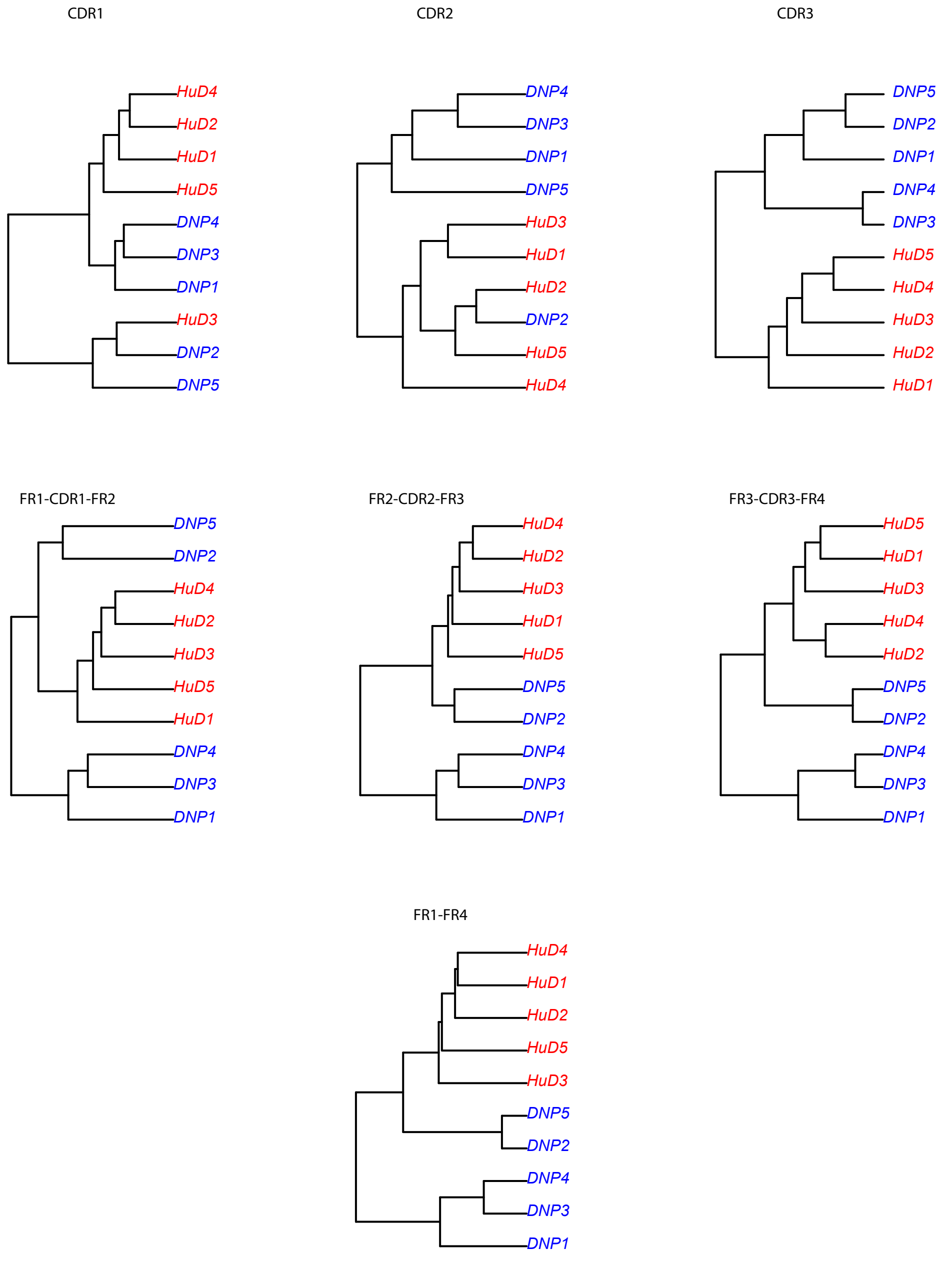

Supplementary Figure 2

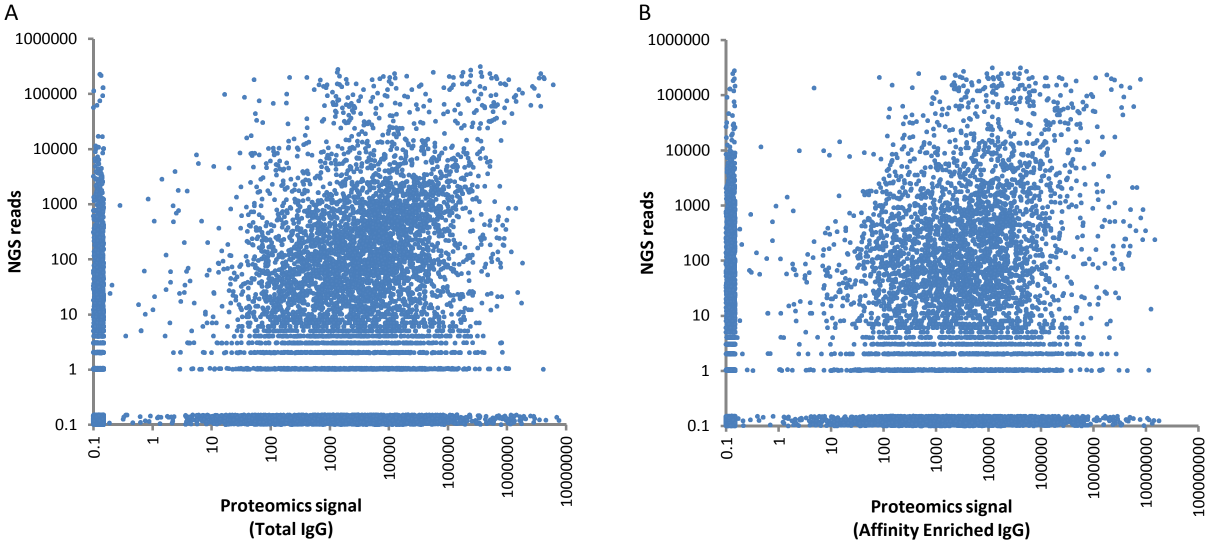

Supplementary Figure 3

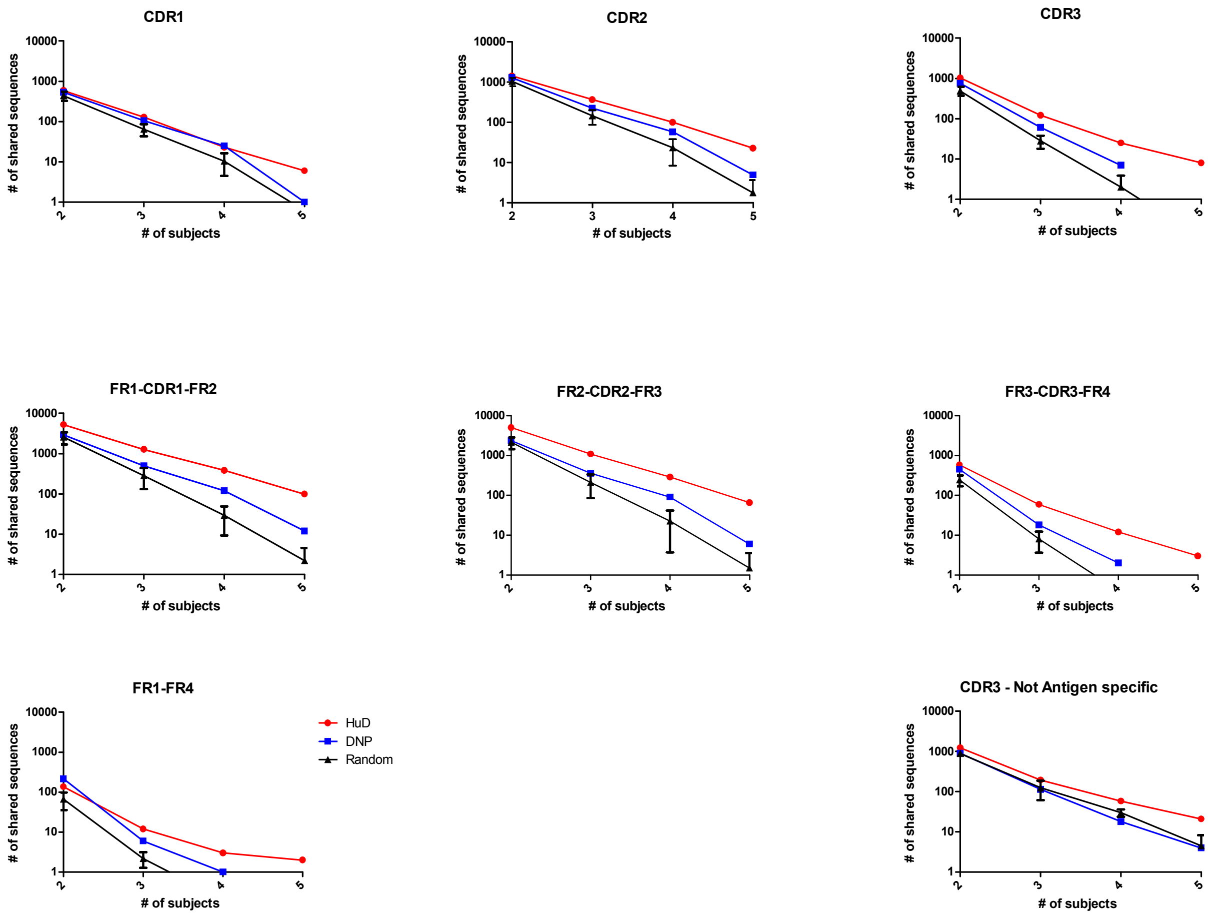

Supplementary Figure 4

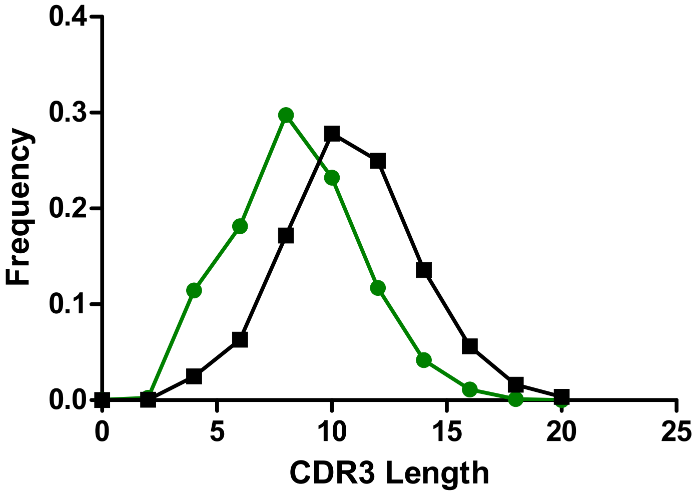

Supplementary Figure 5

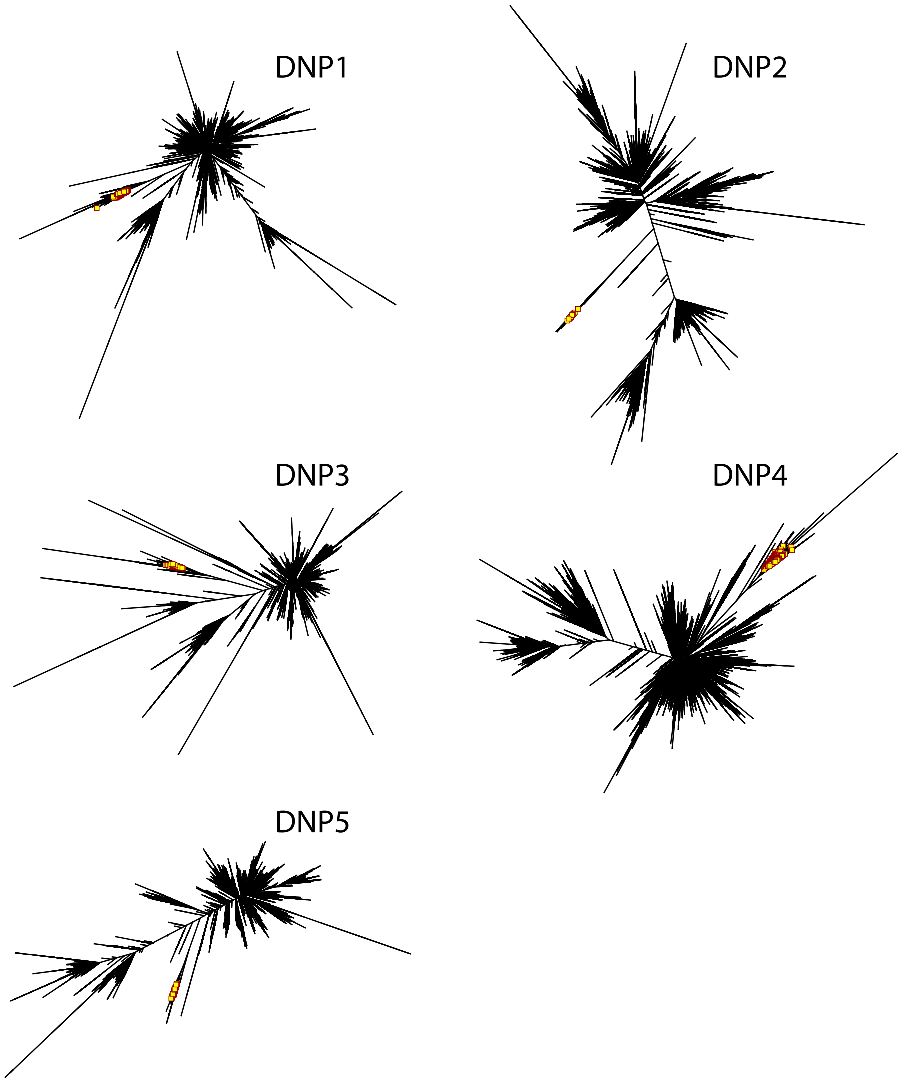

Supplementary Figure 6

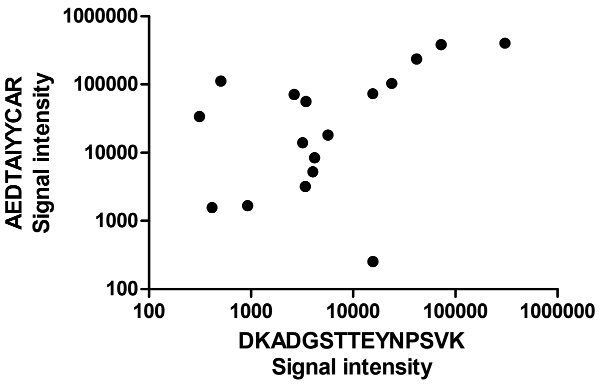

EVKLLESGGGLVQPGGSLRLSCAASGFAFTDFYMS
WIRQPPGKAPEWLGVIR**<u>DKADGSTTEYNPSVK</u>**GRFT
ISRDNTQNIFYLQMNTLR**<u>AEDTAIYYCAR</u>**WTLTTRGF
DYWGQGVMVTVSSAETTAPSV

Supplementary Figure 7

Antigen	Animal	Aff. Enriched, Immunized Spectra	PSM	Unique	Total IgG, Immunized Spectra	PSM	Unique	Total IgG, Pre-immune Spectra	PSM	Unique
HuD	1	5065	197	177	4989	358	302	3947	332	276
	2	5221	202	178	4651	502	388	3936	321	269
	3	4981	231	197	5273	511	365	4071	326	267
	4	5568	210	183	4845	550	393	4091	369	304
	5	5566	180	156	4285	419	328	4018	380	315
DNP	1	4509	177	133	4335	416	346	4348	342	278
	2	5130	172	132	4807	418	333	4029	353	291
	3	4742	209	181	4822	476	382	4058	325	273
	4	5363	328	201	4801	514	415	4091	344	286
	5	4906	137	113	4049	405	344	4826	307	255

Supplementary Table 1

Antigen	Animal	PSM metric	Matched database search	Unmatched database search
HuD	1	Total PSM	190	160
		Matched in both	126	124
		Matched uniquely	64	36
HuD	2	Total PSM	192	156
		Matched in both	143	129
		Matched uniquely	49	27
HuD	3	Total PSM	214	192
		Matched in both	155	159
		Matched uniquely	59	33
HuD	4	Total PSM	199	178
		Matched in both	141	143
		Matched uniquely	58	35
HuD	5	Total PSM	139	142
		Matched in both	106	106
		Matched uniquely	33	36
DNP	1	Total PSM	178	125
		Matched in both	96	97
		Matched uniquely	82	28
DNP	2	Total PSM	190	163
		Matched in both	154	148
		Matched uniquely	36	15
DNP	3	Total PSM	232	179
		Matched in both	154	146
		Matched uniquely	78	33
DNP	4	Total PSM	249	165
		Matched in both	143	142
		Matched uniquely	106	23
DNP	5	Total PSM	121	117
		Matched in both	91	92
		Matched uniquely	30	25

Supplementary Table 2

m/z	Sequence	Immunogen	Number of Reads									
			HuD1	HuD2	HuD3	HuD4	HuD5	DNP1	DNP2	DNP3	DNP4	DNP5
697.7918	SEDTAMYYCVR	DNP	5	14	4	10	2	1	6	1	5	15
	SEDMATYYCVR	DNP	3	2	11	50	1063	46962	4	5659	5253	1856
878.9334	ASGYPFTSHYISWIK	DNP	3	0	0	0	0	0	0	0	0	0
	ASGYTFTGYYISWIK	DNP	0	2	2	0	1	1	0	0	2	0
	ASGYTFTSYYIGWIK	DNP	0	11	0	1	5	975	43	460	3896	1239
	ASGYTFTSYYIGWLK	DNP	0	0	0	0	0	23	1	6	39	1
	ASGYTFTSYYLGWIK	DNP	0	0	0	0	0	7	11	5	36	1

Supplementary Table 3
